# The association between self-perceived stress and ischemic stroke risk: a systematic review and meta-analysis

**DOI:** 10.3389/fneur.2025.1605470

**Published:** 2026-01-06

**Authors:** Yanyan Li, Bo Wang, Peng Gao, Ziqi Liu, Ying Xu, Xiaorui Pei

**Affiliations:** 1 Department of Neurology Ward, Chaoyang Central Hospital of China Medical University, Chaoyang, Liaoning, China; 2 Department of Reproductive Medicine, Chaoyang Central Hospital of China Medical University, Chaoyang, Liaoning, China; 3 Xinhua Clinical College, Dalian University, Dalian, Liaoning, China

**Keywords:** self-perceived stress, stroke, mortality, stress, risk factors

## Abstract

**Background:**

An increasing body of research indicates that psychological stress is a contributing factor to stroke. Nonetheless, the correlations between self-perceived stress and stroke remain ambiguous. We performed the first meta-analysis on the correlation between self-perceived stress and stroke risk, establishing a clear relationship between self-perceived stress and stroke.

**Methods:**

Two reviewers independently searched electronic databases (MEDLINE, PubMed, Cochrane Library, Web of Science, and EMBASE database) for stroke and self-perceived stress studies. Studies employing the Perceived Stress Scale (PSS), Single question, or 2 single-item questions assessment tools were included, studies were executed and presented in English from inception to March 7, 2025. Eleven papers were included into this meta-analysis.

**Results:**

(1) In our meta-analysis, the multivariable-adjusted relative risk (RR) indicated that self-perceived stress was independently associated with stroke risk. (2) Subgroup analysis revealed that individuals with high self-perceived stress had a significantly elevated stroke risk, whereas no significant association was observed in those with low self-perceived stress. (3) Furthermore, our meta-analysis demonstrated that elevated self-perceived stress was associated with higher stroke mortality; (4) In sex-specific analysis, self-perceived stress was significantly associated with increased stroke risk in women, but not in men.

**Conclusion:**

Self-perceived stress was associated with increased stroke risk, especially in individuals reporting moderate-high self-perceived stress levels and women. Furthermore, elevated self-perceived stress was correlated with stroke mortality.

**Systematic review registration:**

PROSPERO, CRD420251026081.

## Introduction

Stroke is the leading cause of death and functional impairment in China (1), which significantly impairs patients’ quality of life and places a substantial burden on both families and society. There is an increasing scientific interest in investigating understudied stroke risk factors, as conventional vascular risk factors alone cannot adequately account for stroke etiology (2, 3). Therefore, identification of more modifiable risk factors to further improve stroke prevention is of great importance.

Psychological stress, stemming from adversity, is a significant factor in the onset of disease. An increasing amount of researches indicated that psychosocial variables and notably psychological stress contribute to cerebrovascular disease (4). Stress may be a contributing factor for ischemic stroke in modern society, particularly in patients without classical risk factors. The stress owing to increasing demands and pressures of employment, such as extended hours, job instability, and elevated expectations. Furthermore, familial issues and financial obligations might substantially contribute to chronic stress (5). The link between stress and cerebrovascular disease may operate through both direct and indirect mechanisms. Chronic psychological stress heightens sympathetic nervous system activity, contributing to elevated blood pressure, tachycardia, insulin resistance, enhanced platelet aggregation, and endothelial dysfunction (6). Chronic stress exerts long-term effects on cerebrovascular, metabolic, and immune regulation, promoting atherosclerosis progression (7). While numerous studies have investigated the link between psychological stress and stroke risk, their inconsistent results underscore the complexity of this relationship (8–12). A Swedish cohort study found that persistent self-reported psychological stress was significantly associated with ischemic stroke, demonstrating a 3.5-fold elevated risk among chronically stressed individuals (10). These findings indicate that chronic stress perception may represent an independent, clinically significant stroke risk factor. A subsequent study (9) corroborated these findings, identifying perceived stress severity, recurrent stressful events, and inadequate coping strategies as independent stroke risk factors. However, conflicting evidence exists. One study (13) reported no significant correlation between perceived stress and stroke incidence.

In summary, while evidence suggests that self-perceived stress may increases stroke risk, the inconsistent findings highlight the need to further examine the relationship between self-perceived stress and stroke. Consequently, we performed the systematic review and meta-analysis to evaluate the association between self-perceived stress and ischemic stroke risk and mortality, another purposes of the study was to examine whether different levels of self-perceived stress were associated with stroke, to provide high-quality, evidence-based recommendations for clinicians in stroke management.

## Materials

### Search strategy and study selection

A thorough literature search was performed from conception to March 7, 2025, employing the MEDLINE, PubMed, Cochrane Library, Web of Science, and EMBASE databases. The following search phrases and keywords were linked by “and” or “or”: ((“Stress, Psychological”[Mesh]) OR (self-perceived stress) OR (perceived stress) OR (psychological stress) OR (mental stress)) AND ((“Stroke”[Mesh]) OR (stroke) OR (cerebrovascular accident) OR (brain infarction)) AND ((“Mortality”[Mesh]) OR (mortality) OR (death) OR (survival) OR (fatality)) AND ((“Risk Factors”[Mesh]) OR (risk factor*) OR (predictor) OR (association)). The study was executed in compliance with the PRISMA (11) standard and was preregistered in PROSPERO (see the Supplementary file-PROSPERO, CRD420251026081).

## Methods

### Data extraction and study quality

#### Inclusion criteria

The search strategy was confined to publicly accessible data and publications in English. Publications were selected according to the following criteria: (1) prospective cohort, cross-sectional, or case–control study; (2) Research investigating the association between self-perceived stress and stroke; (3) Outcome: Eligibility for meta-analysis required studies to provide risk estimates with adjustment for a minimum of three established stroke risk factors. (4) A comprehensive definition of stroke was established, encompassing ischemic stroke; (5) The definition of self-perceived stress used were a single-item questionnaire (12) (In this questionnaire stress is described as feeling tense, irritable, anxious, or as having sleeping difficulties as a result of conditions at home or at work. Participants were asked to report how often they had felt stress, using the following incremental or graded response options) and the 10-item Perceived Stress Scale (13) (PSS10, PSS is a widely recognized questionnaire that consists of 10 questions designed to evaluate the degree to which individuals perceive their lives as stressful); (6) For the questions on self-perceived stress, participants were asked to indicate the frequency of their stress using the following response options: 1: never; 2: some periods; 3: several periods; or 4: permanent stress. (7) Classification Criteria for self-Perceived Stress Groups: (1) For the purpose of subgroup analysis, the PSS-10 scores were dichotomized: Low stress group: PSS-10 score ≤ 13; Medium/High stress group: PSS-10 score > 13. This cut-off point is derived from Cohen’s (1988) normative data, where a score above 13 falls above the population mean, indicating elevated stress levels; (2) Specifically, for a single-item question asking participants to rate their stress on a scale from 0 (‘no stress’) to 10 (‘extreme stress’), the responses were dichotomized as follows: Low stress: Scores ranging from 0 to 3. Medium/High stress: Scores ranging from 4 to 10. (3) Two single-item questions were used to assess stress, a cut-off point was established: Low stress group: A total score of ≤ 3. Medium/High stress group: A total score of ≥ 4 (Table 1).

**Table 1:** Classification criteria for self-perceived stress groups.

Stress level	**Single-item question**	**Two single-item questions**	PSS
Low stress	0–3	0–3	0–13
Medium/High stress	4–10	4–8	14–40

#### Exclusion criteria

Prior to data extraction, two reviewers (Bo Wang and Peng Gao) independently utilized the preliminary data extraction form on a random sample of five studies. The results from both reviewers were then cross-checked, leading to a collaborative discussion and subsequent revision of entries that exhibited inconsistencies or lacked clarity in their definition until a consensus was achieved. Two reviewers, independently extracted data from relevant research utilizing data extraction forms. Discrepancies between the two reviewers were resolved by discussion with a third author (YY Li). We excluded: (1) duplicate or irrelevant papers; (2) reviews, letters, case reports, and comments; (3) non-original research; (4) studies involving non-human subjects; (5) unpublished or non–peer-reviewed studies.

#### Data extraction

Two independent reviewers utilized a standardized data collecting form to retrieve essential data and information from the qualifying studies. This includes the following elements: the author’s name, the publication year of the study, study populations including sex and age, forms of stress exposure and assessment methods, the case and control groups, levels of self-perceived stress, and death rates. The effect estimates and their 95% confidence intervals were derived after accounting for the maximum number of confounding variables.

#### Quality assessment

The two reviewers, Bo Wang and Peng Gao, independently assessed the quality and risk of bias of the included studies using the Newcastle-Ottawa Scale (NOS) (14); All disagreements between the two reviewers were extensively assessed, and moreover, a third reviewer (YY Li) was assigned to resolve any residual discrepancies and attain a consensus.

### Statistical analysis

The meta-analysis used STATA version 17.0. A meta-analysis was conducted to aggregate adjusted risk estimates from the studies that reported them. In cohort studies, hazard ratios (HRs) served as the standard risk measure across research, with relative risks deemed comparable to HRs. In case–control studies, odds ratios (ORs) served as the standard risk measure. If many adjusted risk estimates were presented, the most comprehensively adjusted estimate was included. Forest plots were generated to visually evaluate the connection among the listed research. The potential for publication bias was assessed by visual examination for any skewness in a funnel plot and Egger’s test. Sub-group analyses were conducted based on gender and levels of self-perceived stress exposure. Sensitivity analyses were conducted to examine the effects of certain research features. When significant heterogeneity exists (*I*^2^ ≥ 50%), sensitivity analyses should be conducted through sequential study exclusion and subgroup analyses to verify result stability. Statistical significance was determined by *p*-values < 0.05, along with 95% confidence intervals. Heterogeneity among studies was assessed using Cochrane’s *Q* test and the *I*^2^ statistic. The *I*^2^ statistic was used to quantify the degree of heterogeneity, with values of 25, 50, and 75% typically indicating low, moderate, and high heterogeneity, respectively. The choice between a fixed-effect and random-effects model was based on the heterogeneity assessment. Data followed by *p* < 0.05 or *I*^2^ > 50% were considered to denote statistically significant heterogeneity, and were subjected to a randomized-effects model. Otherwise, if *I*^2^ < 50% a fixed-effects model was used.

## Results

### Study characteristics

#### Literature search and study characteristics

**Figure 1:**
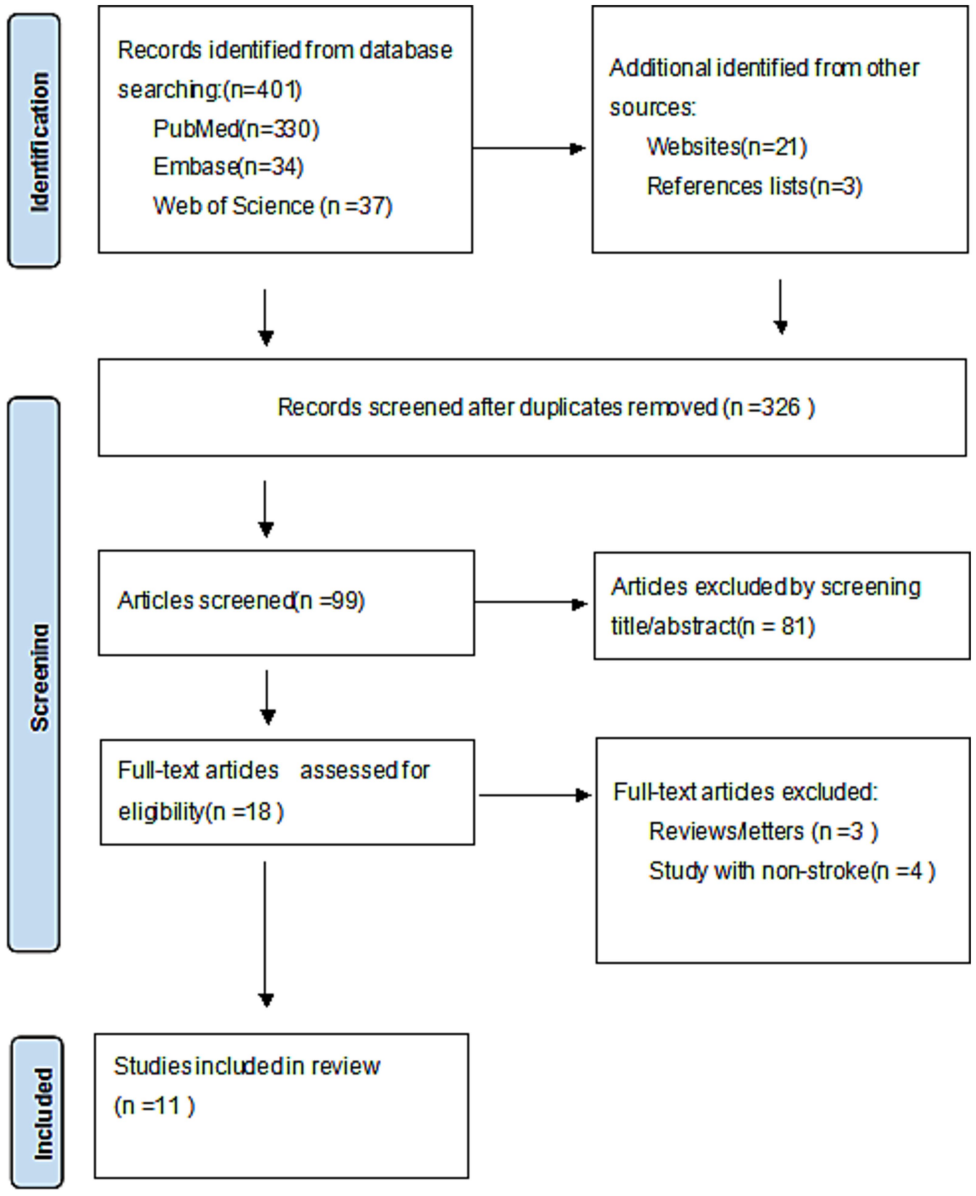
Preferred Reporting Item for Systematic Reviews and Meta-Analysis (PRISMA) guideline.

Our initial search identified a total of 401 records from MEDLINE, PubMed, Cochrane Library, Web of Science, and EMBASE databases and other sources: 3 records from reference lists and 21 records from websites. After removing 326 duplicates, 99 unique records remained for title and abstract screening. During the title and abstract screening phase, 81 records were excluded as they clearly did not meet the inclusion criteria. The full text of the remaining 18 articles was thoroughly assessed for eligibility. Three reviews and four non-stroke studies were excluded (Figure 1). Consequently, 11 studies with 247,873 persons examining the association between self-perceived stress and stroke were included for qualitative synthesis, followed by meta-analysis (Table 2). The quality Table 2 outlines the main attributes of the included research, and Figure 1 provides a PRISMA-style flowchart of the literature screening process. NOS scores varied between seven and nine (Table 2). All included records were assessed to be of exceptional quality.

**Table 2:** Characteristics of the Included Studies.

Study and year	Study country	Cohort size	Stress exposure and measure	follow-up duration	OR/RR for stroke	Con-founders adjusted for	population characteristics	NOS
Li 2021 (17)	China	20,688	self-perceived psychological stress Single question	followed up every 3 months for 4.5 years	Medium 1.11 (0.91, 1.35) High 1.45 (1.01, 2.09)	Adjusted for treatment group, age, sex, BMI, study centers, smoking, alcohol consumption, living standard, fasting glucose, total homocysteine, total cholesterol, triglycerides, high-density lipoprotein cholesterol, creatinine, SBP, DBP, and mean SBP and DBP, the use of calcium channel blockers and diuretics during the treatment period	Hypertensive adults in 32 communities in Jiangsu and Anhui provinces in China	8
Harmsen 2006 (7)	Sweden	7,457	Self-perceived stress Single question	0–15, 16–21, and 22 to 28 years of follow-up	1.25 (1.03–1.51)	Adjusted for SBP, Previous TIA, AF, Stroke, History of diabetes, Coronary events, Smoking History, BMI, Low physical activity	A cohort of 7,457 men 47–55 years of age and free of stroke at baseline year 1970 were examined	8
Jood 2009 (4)	Sweden	1,200	self-perceived stress Single question	Follow up visit during the acute stage and after 3 months	3.49 (2.06, 5.93)	adjusted for age, sex, hypertension, smoking status, diabetes, hyperlipidemia, occupation, leisure time physical activity, waist to hip ratio and family history of stroke	The study population includes the participants in the SAHLSIS cohort. In short, Caucasian patients presenting with acute ischemic stroke before the age of 70 years were consecutively recruited from four stroke units in Western Sweden	7
Kutal 2025 (19)	Finland	852	self-perceived stress Perceived Stress Scale (PSS)	within 12 months preceding the stroke	Medium: 1.47 (1.00, 2.14) High: 2.62 (0.81, 8.45)	Adjusted for age, level of education, predefined vascular risk factors, and migraine with aura	Young patients aged 18–49 years with a first-ever CIS and sex-matched and age-matched stroke-free controls from 19 European centers were included	8
Ramírez-Moreno 2017 (18)	Spain	100	self-perceived stress Single question	within 12 months preceding the stroke	2.33 (1.02, 5.30)	Adjusted for Precarious jobs, seasonal jobs, training jobs, family workers, auxiliary workers, self-employed	The target population consisted of individuals who were economically active and always under 70 years of age, and who had experienced a first episode of cerebrovascular event. Cases were selected from the same population base as the controls, with absolute certainty that they had not suffered any prior vascular disease	7
Gallo 2014 (10)	American	5,313	self-perceived stress Perceived Stress Scale (PSS)	9 months of follow up	1.26 (1.03, 1.55)	Adjusted for conceptually relevant sociodemographic covariates including age, sex, education, income, language of interview, nativity/immigration, and Hispanic/Latino background, BMI, physical activity, alcohol, and smoking and was calculated for all outcomes	Participants were 5,313 men and women, 18–74 years old, representing diverse Hispanic/Latino ethnic backgrounds, who underwent a comprehensive baseline clinical exam and sociocultural exam with measures of stress	8
Truelsen 2003 (8)	Denmark	12,574	Self-perceived stress 2 single-item questions	13 years of follow-up	Fatal stroke: 1.89 (1.11, 3.21) Non-fatal stroke: 1.13 (0.85, 1.50) Low: 0.96 (0.82, 1.13) Medium: 1.10 (0.91, 1.33) High: 1.13 (0.85, 1.50)	Body mass index, smoking, education, physical activity, alcohol, systolic blood pressure, anti-hypertensive treatment	A prospective observational study that was initiated in 1976 when 19,698 subjects living in Østerbro and Nørrebro in Copenhagen were invited to participate in the first study examination	8
Santosa 2021 (25)	Sweden	118,706	Self-perceived stress 2 single-item questions	follow-up of 10.2 years	Fatal stroke: 1.09 (1.03, 1.16) Low: 1.0 (0.96, 1.15) Medium: 1.07 (0.94, 1.21) High: 1.30 (1.09–1.56)	Adjusted for age, education, marital status, location, abdominal obesity, hypertension, smoking, diabetes, family history of CVD, and center random effects	This population-based cohort study used data from the Prospective Urban Rural Epidemiology study, collected between January 2003 and March 2021. Participants included individuals aged 35–70 years living in 21 low-, middle-, and high-income countries	9
Rosengren 1991 (15)	Sweden	6,935	Self-perceived stress 2 single-item questions	11.8 year follow-up	1.1 (1.8, 2.8)	Adjusted forage, systolic blood pressure, serum cholesterol, smoking, body mass index, diabetes, family history of myocardial infarction occupational class, marital state, leisure time physical activity and alcohol abuse	The study population of the first part of this study comprises the intervention group of the Multifactor Primary Prevention Trial in Goteborg	7
Mokhber 2020 (16)	Iran	624	Self-perceived stress 2 single-item questions	1-year of follow up	Non-fatal Stroke: 0.86 (0.58, 1.27) Fatal stroke: 0.75 (0.47, 1.20)	Adjusted for age, sex, socio-economic variables, atrial fibrillation, hypertension, diabetes mellitus and hyperlipidaemia	Patients were recruited from the Mashhad Stroke Incidence Study (MSIS), a population-based stroke study in Mashhad, Iran	7
Iso 2002 (9)	Japan,	73,424	Self-perceived mental stress Single question	follow-up period was 7.9 years	Fatal stroke: 1.33 (0.81, 2.19)	Adjusted for sex, age, body mass index, smoking status, alcohol intake, hours of walking, hours of sleep, and psychological variables, including anger, hurry, self-estimation of quick response, hopelessness, senses of joyfulness, being trusted, fulfillment among full-time workers, job stress and control	From 1988 to 1990, a total of 73,424 Japanese (30,180 men and 43,244 women), aged 40–79 years, without a history of stroke, CHD, or cancer completed a lifestyle questionnaire including perception of mental stress under the Japan Collaborative Cohort Study for Evaluation of Cancer Risk Sponsored by Monbusho (JACC Study)	8

PSS, Perceived Stress Scale.

#### Self-perceived stress and risk of stroke

**Figure 2:**
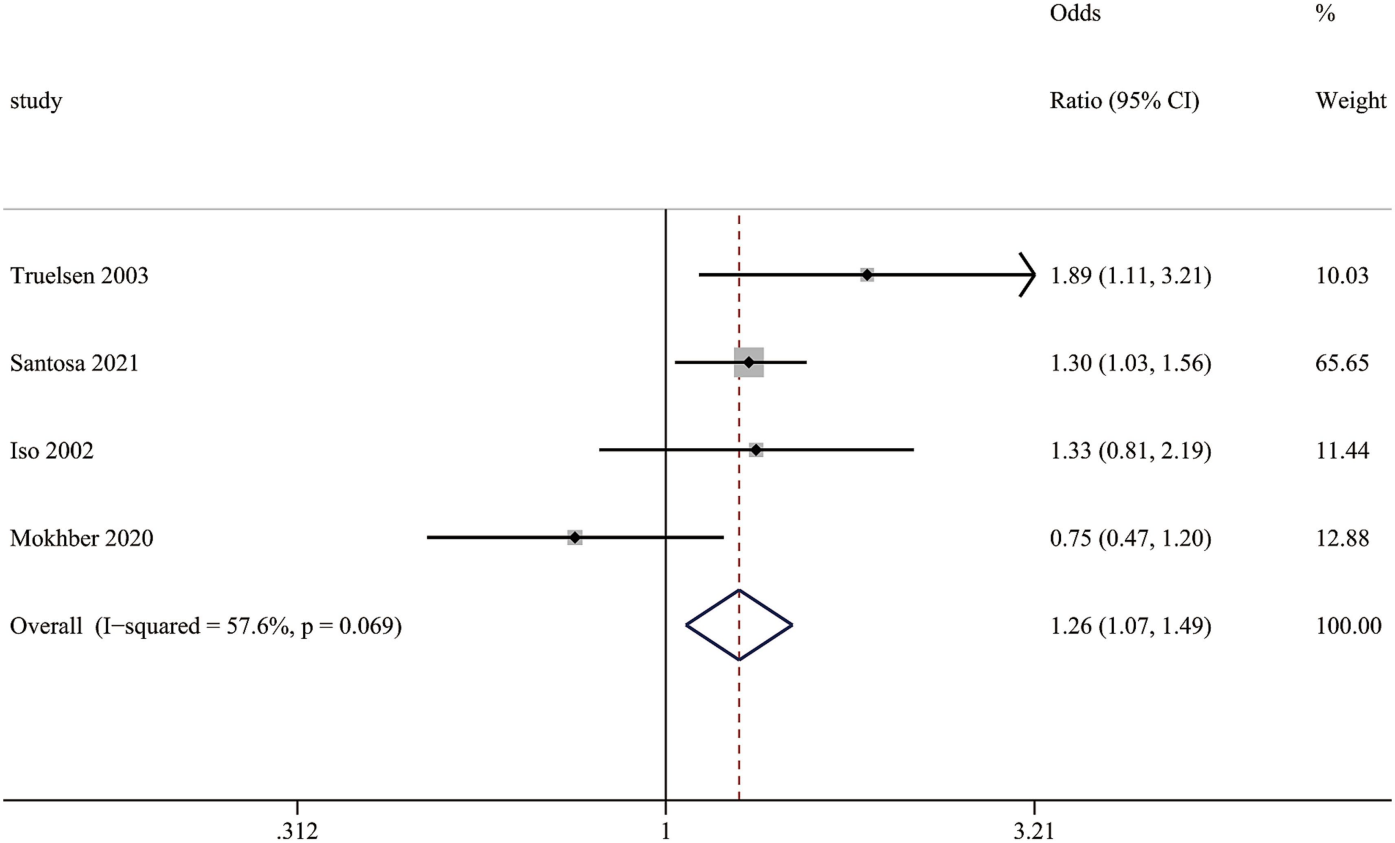
Association between self-perceived stress and risk of stroke.

In comparison to persons without self-perceived stress, the multivariable-adjusted relative risk (RR) of stroke for those with self-perceived stress was (1.04 (1.02, 1.06), *I*^2^ = 62.8%) (Figure 2), which demonstrated that individuals with self-perceived stress had an elevated risk of stroke.

#### Subgroup analysis of multivariable-adjusted relative risk (RR) and sensitivity analysis in ischemic stroke and self-perceived stress

We performed subgroup and sensitivity analyses based on the type of effect measure and the specific self-perceived stress scale used. The results stratified by effect measure, were as follows: for studies reporting Odds Ratios, the multivariable-adjusted RR was 1.31 (95% CI: 1.16, 1.48) with an *I*^2^ of 79.1%; for those reporting Risk Ratios, the multivariable-adjusted RR was 1.34 (95% CI: 1.06, 1.70) with an *I*^2^ of 57.0%; and for studies reporting Hazard Ratios, the multivariable-adjusted RR was 1.29 (95% CI: 1.09, 1.52). The conclusion from this analysis remained consistent with our initial findings, indicating a significant association between self-perceived stress and stroke (Table 3).

**Table 3:** Subgroup analysis of RR and sensitivity analysis in ischemic stroke and self-perceived stress.

Outcome	Studies	RR	95%CI	*I*^2^ for Heterogeneity	*p* for heterogeneity
Effect estimates	10				
OR	5	1.31	1.16, 1.48	79.1%	*p* = 0.001
RR	3	1.34	1.06, 1.70	57%	*p* = 0.098
HR	2	1.29	1.09, 1.52	22%	*p* = 0.000
Assessment scale	10				
PSS-10	2	1.29	1.05, 1.57	31.2%	*p* = 0.228
A single-item question	4	1.43	1.23, 1.67	78.9%	*p* = 0.003
Two single-item questions	4	1.23	1.08, 1.41	53.6%	*p* = 0.091

Furthermore, subgroup analysis based on the self-perceived stress assessment scale also yielded consistent results: the multivariable-adjusted RR was 1.29 (95% CI: 1.05, 1.57) for the group using the Perceived Stress Scale (PSS), 1.43 (95% CI: 1.23, 1.67) for the group using a single-item question, and 1.23 (95% CI: 1.08, 1.41) for the group using two single-item questions. This confirms that the association between self-perceived stress and stroke persists regardless of the measurement tool used (Table 3).

#### The multivariable-adjusted relative risk (RR) of stroke for different levels of self-perceived stress

**Figure 3:**
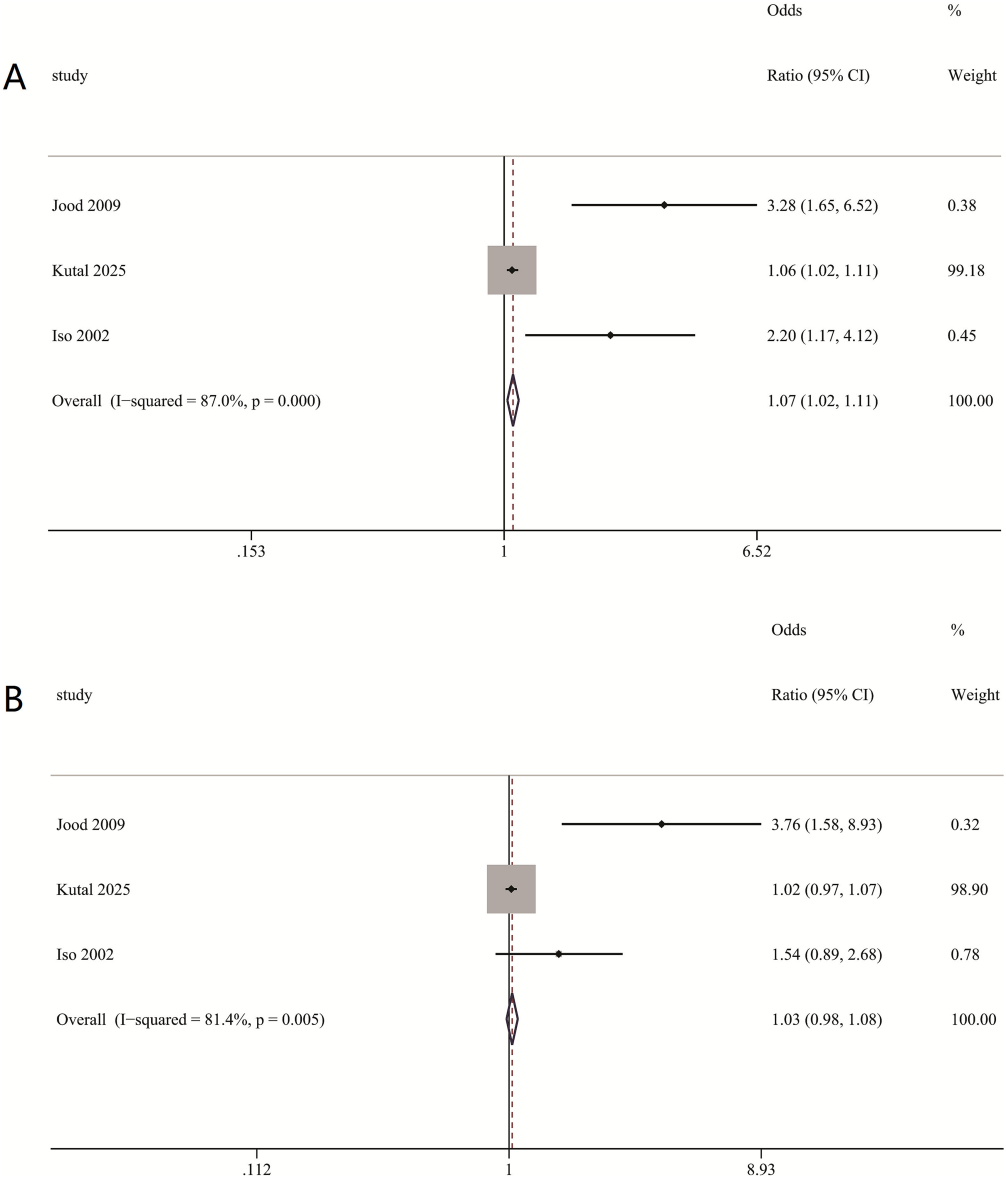
Multivariable-adjusted relative risks of stroke according to levels of self-perceived stress. **(A)** Association between low levels of self-perceived stress and stroke risk. **(B)** Association between medium-to-high levels of self-perceived stress and stroke risk.

The subgroup analysis indicated that medium-to-high levels of self-perceived stress are independently linked to a heightened risk of stroke, RR = (1.31 (1.14, 1.50), *I*^2^ = 9.6%) (Figure 3B), whereas low levels of self-perceived stress exhibit no significant association with stroke, RR = (1.08 (0.99, 1.18) *I*^2^ = 7.6%) (Figure 3A). This suggests a potential threshold effect, where the detrimental impact on stroke risk becomes clinically relevant beyond a certain stress intensity. The consistently low heterogeneity (*I*^2^ = 7.6–9.6%) underscores the robustness of this differential association across diverse study settings.

#### Association between self-perceived stress and the risk of stroke mortality

**Figure 4:**
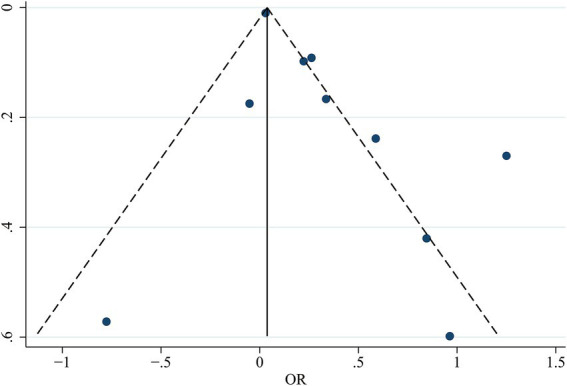
Funnel plot for the assessment of potential publication bias.

This meta-analysis reveals a correlation between self-perceived stress and stroke mortality. Patients experiencing self-perceived stress have a considerably higher mortality compared to those without stress, with relative risk (RR) of (1.26 (1.07, 1.49), *I*^2^ = 57.6%) (Figure 4). Despite moderate heterogeneity (*I*^2^ = 57.6%), the robust effect size underscores stress as an important prognostic factor in stroke outcomes.

#### Association between self-perceived stress and incident stroke, stratified by sex

**Figure 5:**
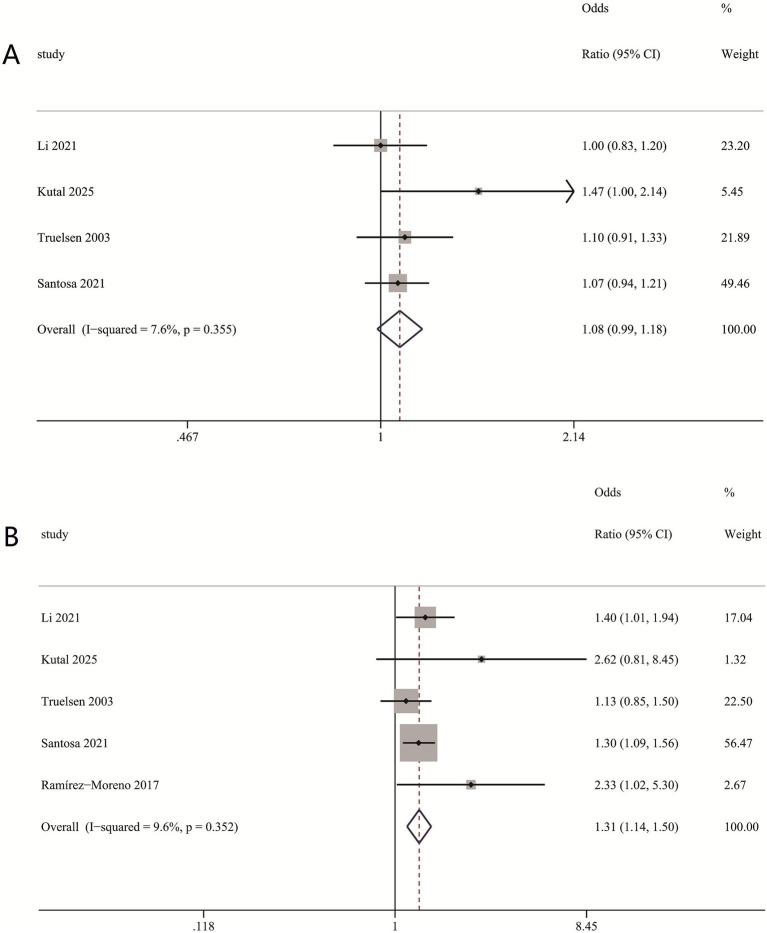
Association between self-perceived stress and incident stroke, stratified by sex. **(A)** Associations between self-perceived stress and risk of incident stroke in women. **(B)** Associations between self-perceived stress and risk of incident stroke in men.

In sex-specific analyses, a significant association between self-perceived stress and stroke was observed in women (RR = 1.07, 95% CI: 1.02–1.11) (Figure 5A), but not in men (RR = 1.03, 95% CI: 0.98–1.08) (Figure 5B). The considerable heterogeneity in both groups (*I*^2^: Women = 87%, Men = 81.4%) suggests underlying variations; however, the positive finding in women, despite this heterogeneity, underscores a sexually dimorphic pattern in the stress-stroke relationship.

### Publication bias

**Figure 6:**
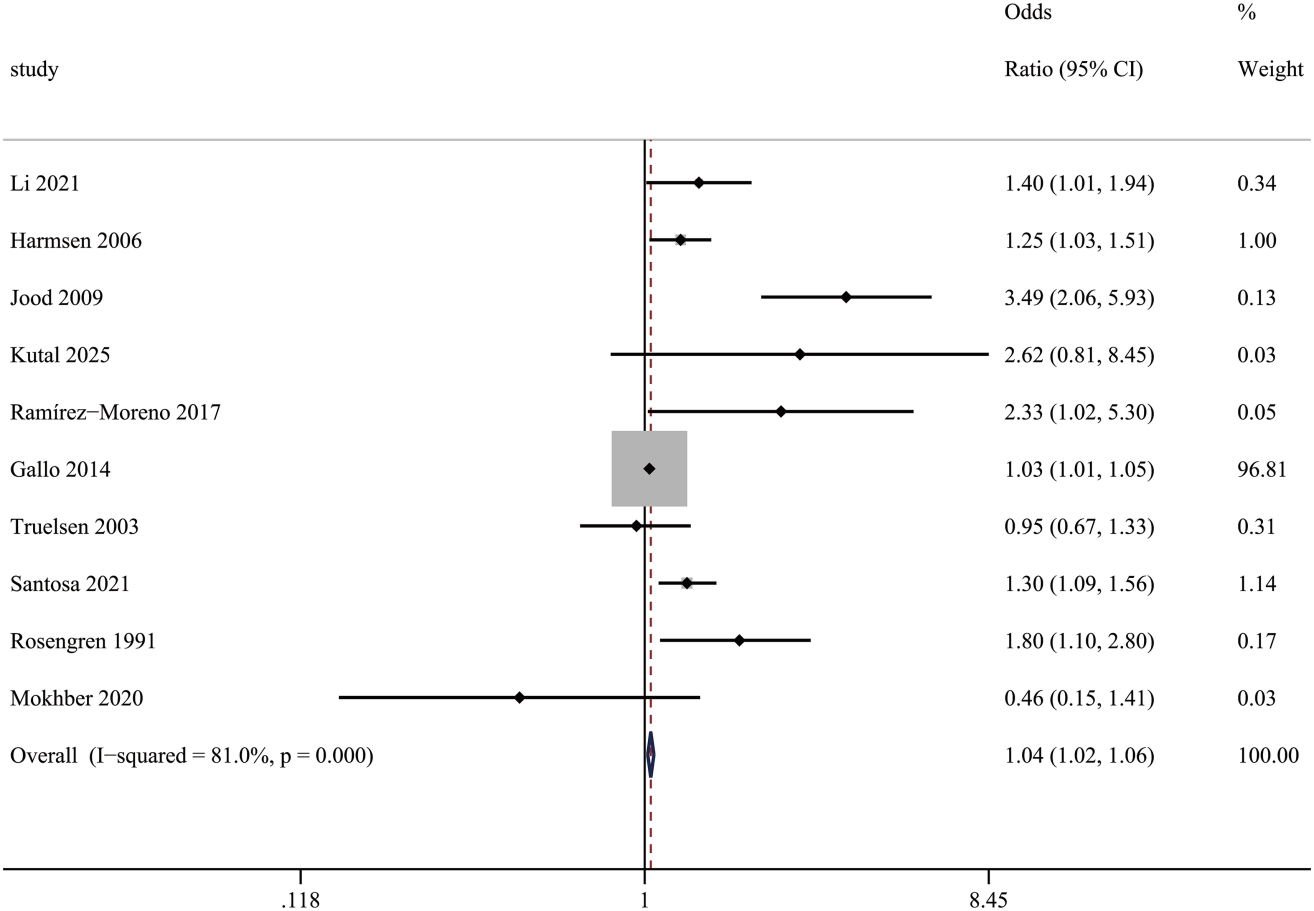
Association between self-perceived stress and the risk of stroke mortality.

The symmetrical appearance of the funnel plot showed no evidence of significant publication bias (Figure 6). Egger’s test results showed a bias coefficient of 1.85 (SE = 1.01, *p* = 0.104) in the regression model, indicating that the findings of this systematic review were not significantly influenced by publication bias or small-study effects.

## Discussion

The present meta-analysis identified a positive correlation between self-perceived stress and stroke risk, indicating that self-perceived stress may serve as an independent risk factor for stroke. Our meta-analysis indicates that those with medium-to-high self-perceived stress levels had a markedly elevated risk of stroke incidence when compared to individuals with low levels of self-perceived stress. The data indicate that heightened self-perceived stress is associated with an elevated risk of stroke. The study concurrently establishes that self-perceived stress correlates with stroke mortality.

Previous research have investigated the correlation between self-perceived stress and stroke across various areas and groups, yielding conflicting results. A body of researches, including studies by Rosengren et al. (15) and Gallo et al. (10), have consistently suggested an association between self-perceived stress and stroke risk. Furthermore, investigations by Jood et al. (4) and Harmsen et al. (7) have refined this understanding by specifying that this relationship holds independently for ischemic stroke. These cumulative findings posit self-perceived stress as a clinically relevant psychosocial risk factor, moving beyond mere correlation to suggest a potential independent role in stroke pathogenesis. Nevertheless, the literature remains inconsistent as evidenced by studies from Mokhber et al. (16) and Truelsen et al. (8), who found no significant association. The persistent contradiction between these sets of findings underscores the need for a comprehensive, quantitative synthesis to clarify the true nature of this relationship, which is a primary objective of the present meta-analysis. Our meta-analysis substantiates the proposition by Harmsen et al. (7) by demonstrating that self-perceived stress is significantly associated with an elevated risk of stroke. Further refining this association, some studies have explored the association between self-perceived stress severity and stroke. Li et al. (17) proved that low self-perceived stress was not correlated with stroke, but high self-perceived stress was recognized as a major risk factor for stroke. This association was confirmed by Ramírez-Moreno et al. (18) study, which found that higher self-perceived stress were related to a higher risk of stroke. Although high self-perceived stress is established as a stroke risk factor, inconsistencies exist regarding its impact at specific severity levels. Kutal et al.’s (19) research revealed an independent correlation between moderate self-perceived stress and stroke, but not with higher self-perceived stress. Our current meta-analysis demonstrated an independent correlation between moderate-to-high self-perceived stress and stroke, but not with low self-perceived stress, confirming that the stroke risk is confined to higher self-perceived stress levels, which is contradictory with the research by Kutal et al. (19).

The mechanisms underlying the stress-stroke relationship are multifaceted. (1) Activation of the Sympathetic Nervous System (20): Self-reported stress triggers sympathetic activation and catecholamine release, leading to heightened blood pressure fluctuation and tachycardia—established risk factors for stroke. The relationship identified in our analysis, where moderate-to-high self-perceived stress confers stroke, is consistent with this mechanism, suggesting a cumulative hemodynamic burden that may only reach pathological significance at moderate-to-high stress intensities. (2) Enhanced Platelet Aggregation (21): Psychological stress can induce platelet hyperreactivity and increase aggregation, promoting thrombus formation and elevating the risk of ischemic stroke. The independent association we observed for moderate-to-high stress provides clinical epidemiological support for this pathway, implying that these stress levels are sufficient to induce a pro-thrombotic state relevant to stroke pathogenesis. (3) Endothelial Dysfunction: Chronic stress adversely affects endothelium-dependent vasodilation. Endothelial dysfunction diminishes nitric oxide (NO) bioavailability (22, 23), increases vascular tone, and promotes leukocyte adhesion and platelet aggregation, collectively facilitating atherosclerosis and thrombogenesis (24). Our finding that low-level stress was not associated with stroke risk suggests a potential threshold effect. The sustained or intense physiological disruption associated with moderate-to-high stress may be necessary to initiate or accelerate clinically significant endothelial damage. In summary, our meta-analysis not only confirms an association between self-perceived stress and stroke, particularly at moderate-to-high levels, but also provides a clinical epidemiological framework that strengthens the plausibility of key pathophysiological mechanisms, including sympathetic hyperactivity, platelet activation, and endothelial injury.

Certain researchers are keen to investigate the correlation between self-perceived stress and stroke mortality, nevertheless, their results have been conflicting. Recent research by Santosa et al. (25) established the correlation between self-perceived stress and stroke mortality. Mokhber et al. (16) investigated the correlation between self-perceived stress and stroke mortality, revealing that self-perceived stress was not a risk factor for stroke mortality. This finding contradicts the common belief that stress is a significant risk factor for stroke and its associated mortality. Our meta-analysis aligned with the study of Santosa et al. (25), which corroborates the correlation between self-perceived stress and stroke mortality.

However, the underlying biological mechanisms remain incompletely understood. In a study using a male mouse model, the relationship between stress and stroke was explored through analysis of ischemia-induced Bcl-2 expression (26). The Bcl-2 proto-oncogene predominantly promotes cell survival and inhibits both apoptotic and necrotic cell death. The investigation demonstrated that male mice exposed to pre-ischemic stress exhibited a 70% decrease in Bcl-2 expression following cerebral artery occlusion compared with non-stressed control animals (26). Although the direct translational applicability of these findings to humans remains unconfirmed, and interspecies physiological variations may constrain their generalizability, the results propose two plausible explanations (27): (1) stress may impair intrinsic neuroprotective systems, and (2) unidentified molecular pathways could mediate the association between stress and stroke mortality. Therefore, these preliminary insights obtained from animal models require cautious interpretation, and further investigation is essential to establish whether—and through what mechanisms—similar processes function in human pathophysiology.

Several studies sought to investigate the correlation between self-perceived stress and stroke, performing subgroup analyses categorized by sex. The research by Kutal et al. (19) indicated that moderate self-perceived stress correlated with stroke in women, but not in men. In contrast, the study by Jood et al. (4) revealed differing relationships between self-perceived stress and stroke in men and women. Our current meta-analysis shown that self-perceived stress was associated with stroke incidence in women, but not in men, which is consistent with Kutal et al. (19). Regarding the underlying causes of this sex-based difference, the existing literature suggests several potential explanatory pathways, though these require further validation. Psychosocial research indicates that men and women may differ in their stress coping mechanisms and patterns of stress perception (28). Meanwhile, neuroendocrine studies propose that fluctuations in sex hormone levels may modulate women’s physiological reactivity to stress (29, 30). However, these interpretations still need to be confirmed through well-designed epidemiological investigations.

Our meta-analysis is conducted to examine the association between self-perceived stress and stroke risk, providing additional evidence and clinical insights for diagnosis and treatment. Nonetheless, our analysis possesses some limitations. (1) First, heterogeneity among the included studies arose from variations in study populations, follow-up durations, and instruments used to measure stress. Additionally, the limited number of available studies may affect the generalizability of our findings. Therefore, the results should be interpreted with caution considering these contextual factors. (2) Second, differences in assessment tools and the use of self-report questionnaires, which are susceptible to recall and social desirability biases, may account for the observed heterogeneity and potentially weaken the true effect size. Future studies should employ standardized instruments to yield more robust evidence. (3) Third, in interpreting our findings, a fundamental challenge lies in the considerable overlap and complex interplay between self-perceived stress and traditional vascular risk factors. Although the included studies generally adjusted for established risk factors such as hypertension and smoking in their statistical models, the influence of residual confounding remains substantial. It is well recognized that unmeasured factors—such as socioeconomic status, lifestyle behaviors, and comorbidities—may still affect the observed association, and these factors are themselves potent drivers of vascular disease. Thus, future large-scale studies are needed to account for these confounding factors and to provide more robust evidence regarding the relationship between self-perceived stress and stroke. (4) The systematic review is limited by the predominance of observational studies among the included literature. Consequently, the current evidence does not support definitive causal inferences regarding the relationship between self-perceived stress and stroke. Future research should prioritize randomized controlled trials and prospective cohort studies to establish causality. (5) Our systematic review revealed an association between self-perceived stress and stroke in women, but not in men. This sex-specific difference may be attributed to the limited number of studies conducting sex-stratified analyses, highlighting the need for larger, sex-stratified studies in the future. (6) As our meta-analysis did not incorporate unpublished or non-peer-reviewed studies (gray literature), there is a potential risk of publication bias. Therefore, future studies are warranted to validate our findings.

## Conclusion

In conclusion, this study provides epidemiological evidence suggesting a potential association between self-perceived stress and an increased risk of stroke, particularly among individuals reporting higher stress levels. Self-reported stress was also correlated with stroke mortality. However, these findings should be interpreted with caution in light of significant heterogeneity, potential unmeasured confounding, and limitations in the measurement of stress itself. While it may be reasonable to consider psychological well-being—including self-perceived stress—as part of a comprehensive stroke risk assessment, the current evidence does not firmly support direct clinical interventions targeting stress reduction solely for stroke prevention. Future research with more rigorous stress assessment and causal study designs is needed before translating these results into clinical practice.

## Data Availability

The raw data supporting the conclusions of this article will be made available by the authors, without undue reservation.
