# Supplementary material for: The association between self-perceived stress and ischemic stroke risk: a systematic review and meta-analysis
Source: Front Neurol. 2026 Jan 6;16:1605470. doi: 10.3389/fneur.2025.1605470 (PMC12815757; doi:10.3389/fneur.2025.1605470)
Supplement: Supplementary file 1 [file Supplementary_file_1.pdf]

NIHR | National Institute for Health and Care Research

PROSPERO
International prospective register of systematic reviews

Self-Perceived Stress and Risk of Ischemic Stroke: A Systematic Review and Meta-Analysis

Yanyan Li, Rui Ding, Ziqi Liu, Xiaorui Pei, Lifeng Piao

Citation
Yanyan Li, Rui Ding, Ziqi Liu, Xiaorui Pei, Lifeng Piao. Self-Perceived Stress and Risk of Ischemic Stroke: A Systematic Review and Meta-Analysis. PROSPERO 2025 CRD420251026081. Available from https://www.crd.york.ac.uk/PROSPERO/view/CRD420251026081.

REVIEW TITLE AND BASIC DETAILS

Review title
Self-Perceived Stress and Risk of Ischemic Stroke: A Systematic Review and Meta-Analysis

Condition or domain being studied
Stress; Ischemic Stroke

Rationale for the review
We performed the systematic review and meta-analysis to evaluate the association between self- perceived stress and stroke risk and mortality, another purposes of the study was to examine whether different levels of self-perceived stress were associated with stroke, to provide high-quality, evidence-based recommendations for clinicians in stroke management..

Review objectives
We performed the systematic review and meta-analysis to evaluate the association between self- perceived stress and stroke risk and mortality, another purposes of the study was to examine whether different levels of self-perceived stress were associated with stroke, to provide high-quality, evidence-based recommendations for clinicians in stroke management..

Keywords
Stress; Ischemic stroke; Risk factors

Country
China

ELIGIBILITY CRITERIA

Population

Included
(1) prospective cohort, cross-sectional, or case-control study; (2) self-reported perceived psychosocial stress;

Intervention(s) or exposure(s)

Included
Stress Management

Comparator(s) or control(s)

Included
PICO tags selected: Stroke Prevention

Study design

Only randomized study types will be included.

Context

We performed the systematic review and meta-analysis to evaluate the association between self- perceived stress and stroke risk and mortality, another purposes of the study was to examine whether different levels of self-perceived stress were associated with stroke, to provide high-quality, evidence-based recommendations for clinicians in stroke management..

TIMELINE OF THE REVIEW

Date of first submission to PROSPERO

04 April 2025

Review timeline

Start date: 4 April 2025. End date: 15 April 2025.

Date of registration in PROSPERO

04 April 2025

AVAILABILITY OF FULL PROTOCOL

Availability of full protocol

A full protocol has been written and uploaded to PROSPERO. The protocol will be made available after the review is completed.

SEARCHING AND SCREENING

Search for unpublished studies

Only published studies will be sought.

Main bibliographic databases that will be searched

The main databases to be searched are *Embase - Embase via Ovid*, *MEDLINE*, *PubMed* and *SCI - Science Citation Index*.

Search language restrictions
The review will only include studies published in English.

Search date restrictions
There are no search date restrictions.

Other methods of identifying studies
Other studies will be identified by: *reference list checking* and *searching conference proceedings*.

Link to search strategy
A full search strategy is available in the full protocol as described in the *Availability of full protocol* section

Selection process
Studies will be screened independently by at least two people (or person/machine combination) with a process to resolve differences.

Other relevant information about searching and screening
None

DATA COLLECTION PROCESS

Data extraction from published articles and reports
Data will be extracted independently by at least two people (or person/machine combination) with a process to resolve differences.

Authors will be asked to provide any required data not available in published reports.

Study risk of bias or quality assessment
Risk of bias will be assessed using: *Newcastle-Ottawa*

Data will be assessed independently by at least two people (or person/machine combination) with a process to resolve differences.

Additional information will be sought from study investigators if required information is unclear or unavailable in the study publications/reports.

Reporting bias assessment
Risk of bias due to missing results will be assessed

Certainty assessment
Certainty of findings will not be assessed

OUTCOMES TO BE ANALYSED

Main outcomes
Self-perceived stress and Risk of stroke

The multivariable-adjusted relative risk (RR) of stroke for different levels of self-perceived stress

Additional outcomes

Self-perceived stress and mortality of stroke

Association Between self-perceived stress and stroke according to Sex grouped by sex

PLANNED DATA SYNTHESIS

Strategy for data synthesis

In cohort studies, hazard ratios (HRs) served as the standard risk measure across research, with relative risks deemed comparable to HRs. In case–control studies, odds ratios (ORs) served as the standard risk measure. If many adjusted risk estimates were presented, the most comprehensively adjusted estimate was included. Forest plots were generated to visually evaluate the connection among the listed research. The potential for publication bias was assessed by visual examination for any skewness in a funnel plot. Sub-group analyses were conducted based on gender and levels of self-perceived stress exposure. Sensitivity analyses were conducted to examine the effects of certain research features. Statistical significance was determined by P-values < 0.05, along with 95% confidence intervals.The variability among studies was evaluated using Cochrane-based Q and I^2 tests. Data with p < 0.05 or I^2 > 50% were considered to reflect statistically significant heterogeneity and were analyzed using a random-effects model.

CURRENT REVIEW STAGE

Stage of the review at this submission

Review stage	Started	Completed
Pilot work		
Formal searching/study identification		
Screening search results against inclusion criteria		
Data extraction or receipt of IPD		
Risk of bias/quality assessment		
Data synthesis		

Review status

The review is currently planned or ongoing.

Publication of review results

Results of the review will be published.

REVIEW AFFILIATION, FUNDING AND PEER REVIEW

Review team members

Mrs Yanyan Li (review guarantor). Chaoyang centra hospital. China.

No conflict of interest declared.

Mrs Rui Ding. chaoyang central hospital. China.

No conflict of interest declared.

Miss Ziqi Liu. dalian university. China.

No conflict of interest declared.

Mrs Xiaorui Pei. chaoyang central hospital. China.

No conflict of interest declared.

Mr Lifeng Piao. chaoyang central hospital. China.

No conflict of interest declared.

Named contact

Mr Lifeng Piao (piaolifeng2024@163.com). chaoyang central hospital. China.

Review affiliation

chaoyang central hospital

Funding source

Review has no specific/external funding but is supported by guarantor/review team (non-commercial) institutions.

Peer review

There has been no peer review of this planned review.

ADDITIONAL INFORMATION

Review conflict of interest

Declared individual interests are recorded under team member details.. No additional interests are recorded for this review.

Medical Subject Headings

Humans; Ischemic Stroke; Stress, Psychological; Stroke

SIMILAR REVIEWS

Check for similar records already in PROSPERO

PROSPERO identified a number of existing PROSPERO records that were similar to this one (last check made on 4 April 2025). These are shown below along with the reasons given by that the review team for the reviews being different and/or proceeding.

- The contribution of perceived stress to transient ischaemic attack and stroke in adults: a systematic review [published 18 December 2012] [CRD42012003424]. The review was judged **not to be similar**
- Association between self-perceived stress and stroke in adults： a meta-analysis [published 4 April 2025] [CRD420251012354]. The review was judged **not to be**

similar

- Perceived stress and dietary patterns among university students: a systematic review-meta-analysis [published 29 June 2022] [CRD42022340512]. The review was judged **not to be similar**

PROSPERO version history

- Version 1.0, published 04 Apr 2025

Disclaimer

The content of this record displays the information provided by the review team. PROSPERO does not peer review registration records or endorse their content.

PROSPERO accepts and posts the information provided in good faith; responsibility for record content rests with the review team. The guarantor for this record has affirmed that the information provided is truthful and that they understand that deliberate provision of inaccurate information may be construed as scientific misconduct.

PROSPERO does not accept any liability for the content provided in this record or for its use. Readers use the information provided in this record at their own risk.

Any enquiries about the record should be referred to the named review contact
